# Sodium para-aminosalicylic acid inhibits manganese-induced NLRP3 inflammasome-dependent pyroptosis by inhibiting NF-κB pathway activation and oxidative stress

**DOI:** 10.1186/s12974-020-02018-6

**Published:** 2020-11-17

**Authors:** Dongjie Peng, Junyan Li, Yue Deng, Xiaojuan Zhu, Lin Zhao, Yuwen Zhang, Zhaocong Li, Shiyan Ou, Shaojun Li, Yueming Jiang

**Affiliations:** 1 Department of Toxicology, School of Public Health, Guangxi Medical University, Shuang-yong Road No.22, Nanning, 530021 Guangxi China; 2 Guangxi Colleges and Universities Key Laboratory of Prevention and Control of Highly Prevalent Diseases, Guangxi Medical University, Shuang-yong Road No.22, Nanning, 530021 Guangxi China

**Keywords:** Mn, PAS-Na, Oxidative stress, NLRP3 inflammasome, pyroptosis, NF-κB pathway

## Abstract

**Background:**

The activation of NOD-like receptor protein 3 (NLRP3) inflammasome-dependent pyroptosis has been shown to play a vital role in the pathology of manganese (Mn)-induced neurotoxicity. Sodium para-aminosalicylic acid (PAS-Na) has a positive effect on the treatment of manganism. However, the mechanism is still unclear. We hypothesized that PAS-Na might act through NLRP3.

**Methods:**

The microglial cell line BV2 and male Sprague-Dawley rats were used to investigate the impacts of PAS-Na on Mn-induced NLRP3 inflammasome-dependent pyroptosis. The related protein of the NF-κB pathway and NLRP3-inflammasome-dependent pyroptosis was detected by western blot. The reactive oxygen species and mitochondrial membrane potential were detected by immunofluorescence staining and flow cytometry. The activation of microglia and the gasdermin D (GSDMD) were detected by immunofluorescence staining.

**Results:**

Our results showed that Mn treatment induced oxidative stress and activated the NF-κB pathway by increasing the phosphorylation of p65 and IkB-α in BV2 cells and in the basal ganglia of rats. PAS-Na could alleviate Mn-induced oxidative stress damage by inhibiting ROS generation, increasing mitochondrial membrane potential and ATP levels, thereby reducing the phosphorylation of p65 and IkB-α. Besides, Mn treatment could activate the NLRP3 pathway and promote the secretion of IL-18 and IL-1β, mediating pyroptosis in BV2 cells and in the basal ganglia and hippocampus of rats. But an inhibitor of NF-κb (JSH-23) treatment could significantly reduce LDH release, the expression of NLRP3 and Cleaved CASP1 protein and IL-1β and IL-18 mRNA level in BV2 cells. Interestingly, the effect of PAS-Na treatment in Mn-treated BV2 cells is similar to those of JSH-23. Besides, immunofluorescence results showed that PAS-Na reduced the increase number of activated microglia, which stained positively for GSDMD.

**Conclusion:**

PAS-Na antagonized Mn-induced NLRP3 inflammasome dependent pyroptosis through inhibiting NF-κB pathway activation and oxidative stress.

**Supplementary Information:**

The online version contains supplementary material available at 10.1186/s12974-020-02018-6.

## Introduction

Manganese (Mn) is one of the occupational and environmental toxicants, although it is an essential trace element for normal physiological functions [1]. At present, high concentration Mn exposure predominantly occurs from welding fumes, mining and smelting of Mn ore, drinking-water contaminated with high levels of Mn, air pollution containing the anti-knocking agent methylcyclopentadienyl Mn tricarbonyl (MTM), or manganic insecticide and ephedron (a psychostimulant drug) abusers [2–5]. Some studies have shown that excessive Mn exposure can injure the globus pallidus, cortex, striatum, subthalamic nucleus, etc. [6, 7]. Excessive Mn accumulated in the basal ganglia may induce extrapyramidal motor dysfunction, leading to manganism, which shares similar clinical symptoms with Parkinson’s disease (PD) [8, 9]. Microglia, the primary innate immune monitors in the central nervous system (CNS), make up 10–15% of nerve cells in the CNS under normal conditions [10]. It is primarily involved in maintaining the normal homeostasis of CNS by producing anti-inflammatory and neurotropic factors etc. [11]. Once environmental exotoxins excessively activate microglia, the balance between its protection and pro-inflammatory effects is interrupted [12]. Significantly, excessive Mn exposure or its combination with lipopolysaccharide (LPS) may induce the activation of microglial cells, which then causes neuroinflammation via promoting the secretion of cytotoxic mediators, such as interleukin-1β (IL-1β) and reactive oxygen species (ROS) [13, 14].

It is well known that NLRP3, a NOD-like receptor and a crucial tissue-damage activator of sterile inflammation, can be activated by diverse damage-associated molecular patterns (DAMPs), such as ROS, mtDNA, ATP, and mitochondrial dysfunction [15]. The activated NLRP3 is assembled into a typical inflammatory complex, called as NLRP3 inflammasome which can recruit pro-caspase-1 [16]. The pro-caspase-1 is transferred to cleaved caspase-1 in an autocatalytic process which could regulate the activation of gasdermin D (GSDMD) [15, 17]. GSDMD is activated by cleaved caspase-1, thus exposes its N-terminal domain. It then oligomerizes with 16-symmetric protomer in membranes to form pores resulting in pyroptosis and IL-1β and IL-18 secretion [18–20]. Pyroptosis, a pro-inflammatory and caspase-1-dependent programmed cell death, has been shown to be associated with heavy metal-induced neurotoxicity via the mediation of the NLRP3-caspase-1 inflammasome or other inflammasomes [21–23]. For instance, an in vitro study showed that cadmium exposure induced NLRP3 inflammasome-dependent pyroptosis by mediating the mitochondrial ROS in human umbilical vein endothelial cells [21]. Moreover, Pei et al. demonstrated that the regulation of endoplasmic reticulum stress on NLRP3-inflammasome-dependent pyroptosis points to a critical pathogenic cause for arsenic-induced pancreatic β cells dysfunction [22]. Additionally, various studies indicated that Mn might induce neuroinflammation by impairing mitochondrial dynamics and modulating MAPK, COX-2, NF-κB pathways or NLRP3-CASP1 pathways [24–28]. Significantly, the activation of microglia and NLRP3 inflammasome may be involved in Mn-induced inflammation [9, 14, 29], and ROS may activate the NLRP3 inflammasome [30, 31]. However, whether NLRP3 inflammasome-dependent pyroptosis participates in Mn-mediated neuroinflammation is rarely reported.

Clinically, EDTA chelation is commonly used to treat heavy metal poisoning. In the case of manganism patients, symptoms are only slightly improved with EDTA chelation; symptoms soon return after chelation treatment is suspended [32]. Interestingly, clinical data showed that sodium p-aminosalicylic acid (PAS-Na) has a curative effect on manganism patients [3, 33]. PAS-Na and its metabolites can cross the blood-brain barrier to play a therapeutic effect, while EDTA cannot pass it and limit its therapeutic effects [32, 34]. In vitro studies have confirmed that PAS-Na treatment increased the activity of antioxidant enzymes in Mn-treated primary basal ganglia [35] or hippocampal neurons [36]. Furthermore, results of an in vivo study indicated that PAS-Na would alleviate Mn-induced inflammation [25, 32, 37]. However, its positive effect on the treatment of Mn-induced neuroinflammation needs to be further explored. Therefore, the present study aims to explore the impact of PAS-Na on Mn-induced NLRP3-caspase-1 inflammasome-dependent pyroptosis, combining between in vitro and in vivo studies. We find that Mn can trigger neurotoxicity particularly by oxidative stress, NF-κB pathway activation and NLRP3 inflammasome-dependent pyroptosis. PAS-Na can restore Mn-induced activation of NLRP3 inflammasome-dependent pyroptosis by inhibiting NF-κB pathway activation and oxidative stress.

## Materials and methods

### Animals and experimental design

Male Sprague-Dawley (SD) rats (aged 6 weeks, weighted 130 ± 20 g), specific pathogen-free (SPF), were purchased from the Experimental Animal Center of Guangxi Medical University (SCXKG2014-0002). All animal procedures performed in this study were performed strictly according to the international standards of animal care guidelines and have been authorized by the Animal Care and Use Committee of Guangxi Medical University. The rats were housed under a constant environment (keeping constant temperature and humidity, light/dark cycle) with food and water ad libitum. After being fed adaptively for 1 week, the animals were randomly divided into 8-week and 14-week experimental periods. The former included control, low (L)-Mn, medium (M)-Mn, and high (H)-Mn-treated groups with 12 rats per group. And rats were injected intraperitoneally (i.p.) with either MnCl_2_·4H_2_O (5, 10, and 20 mg/kg, in saline) or saline (0.9% NaCl) once a day, 5 days per week, based on a published protocol [32]. The latter included control, Mn-treated, Mn+ Low (L)-PAS, Mn+ Medium (M)-PAS, Mn+ High (H)-PAS and PAS-Na control (C-PAS) groups with 12 rats per group. The rats in the Mn-treated group, Mn + L-PAS, Mn + M-PAS, and Mn + H-PAS received i.p. injection with 20 mg/kg MnCl_2_·4H_2_O once a day, 5 days per week for 8 weeks, while rats in the control and C-PAS groups received i.p. injection with saline. Then, rats in the Mn + L-PAS, Mn + M-PAS, and Mn + H-PAS and C-PAS groups received subcutaneously (s.c.) injection with 100, 200, 300, and 300 mg/kg PAS-Na, respectively, once a day, 5 days per week for next 6 weeks, while rats in other groups received s.c. injection with saline, based on our previous studies [25, 32]. After Morris water maze test, the rats were anesthetized by i.p. injection of 3.5% chloral hydrate (10 mL/kg) and sacrificed. The brain was quickly extracted, and the basal ganglia were collected.

### Morris water maze test

The learning and memory ability of the treated rats was assessed by Morris water maze test and performed according to established protocols from previous studies [25, 38]. Briefly, the test consists of two parts: training trials for 5 days and spatial probe trials for 1 day. Firstly, the rats entered the water from the east, south, west and north of the Morris water maze to find the platform, respectively. The total swimming distances and escape latency were recorded. If the platform was not found at 90 s, the rat would be guided to the platform. After training trials, the platform was removed for the later trials. The number of rats crossing the platform and swimming speed within 120 s was recorded.

### Immunohistochemistry

The specific operation steps of immunohistochemistry refer to our previous research [25]. Rabbit anti-CD11b (0.75 μg/mL, Thermo, PA5-79532) was used in this tests.

### Immunofluorescence

The sample process is the same as the previous study [25]. Sections were incubated with the following primary antibody at 4 °C overnight: rabbit anti-GSDMS antibody (1:500, abcam, ab219800), mouse anti-IBA1 (1:200, Servicebio, GB12105, China). The sections were then washed with PBS and incubated with anti-rabbit IgG [1:1000, Alexa Fluor® 488 Conjugate, Cell Signaling Technology (CST), #4412S] and Anti-mouse IgG (1:1000, Alexa Fluor® 594 Conjugate, CST, #8890) for 1 h at room temperature. Lastly, the sections were examined under the EVOS fluorescence microscopy imaging system.

### Cell culture

BV2 cells, a type of microglial cell line, were obtained from the China Center for Type Culture Collection (CCTCC). Cells were placed on T25 flasks and cultured in the culture medium which contains 10% fetal bovine serum (FBS, Gibco, USA), 100 IU/mL penicillin, and 100 μg/mL streptomycin and DMEM/F12. Cells were cultured in a humidified incubator (ThermoScientific, USA) of 5% CO2 at 37 °C. The BV2 cells morphology were observed with an inverted microscope (OLYMPUS, Japan)

### Measurement of ROS

BV2 cells were treated with 10 μmol/L 2′,7′-dichlorodihydrofluorescein dictate (DCFH-DA, Beyotime, China) for 1 h at 37 °C in the dark. The detection was performed in strict accordance with the manufacturer’s instructions and previous studies [39, 40]. The fluorescence was visualized by using the EVOS fluorescence microscopy imaging system (Thermo, USA).

Flow cytometry was used to detect ROS production. Firstly, BV2 cells were collected into 15 mL centrifuge tubes and incubated with 10 μmol/L DCFH-DA at 37 °C in the dark for 1 h (mixing cells every 10 min). Subsequently, cells were re-suspended in pre-cooled PBS, washed in pre-cooled PBS three times, and then transferred to 1.5 mL EP tubes for detection by flow cytometry.

### Detection of mitochondrial membrane potential (mt∆Ψm)

Mt∆Ψm was determined by the lipophilic cationic probe JC-1 (Beyotime, China). At the end of treatment, BV2 cells were treated with 10μg/ml JC-1 at 37 °C in the dark for 30 min. The detection was performed in strict accordance with the manufacturer’s instructions and previous studies [41, 42]. The fluorescence was visualized by using the EVOS fluorescence microscopy imaging system.

Flow cytometry was used to detect mt∆Ψm changes. Firstly, BV2 cells were collected into 15 mL centrifuge tubes and treated with 10 μg/ml JC-1 at 37 °C in the dark for 30 min (mixing cells every 10 min). Subsequently, cells were resuspended in staining buffer and detected by flow cytometry after being washed with pre-cooled PBS three times.

### Adenosine triphosphate (ATP) assay

ATP kit (Nanjing Jiancheng Bioengineering Institute, China) was used to assess the intracellular ATP in BV2 cells. Cells were collected and resuspended in boiled PBS. The cells were lysed using an ultrasonic pulverizer. The detection was performed in strict accordance with the manufacturer’s instructions [43]. Protein levels were detected to normalize the results by using the BCA protein assay kit (Multi Sciences, China).

### Lactate dehydrogenase (LDH) assay

LDH, an indicator of cell membrane integrity, has been shown to indirectly relate to the occurrence of pyroptosis [44]. For this assay, the culture medium was collected and centrifuged at 2500×*g*, 4 °C for 10 min. Then, the supernatant was transferred into 1.5 mL EP tubes. LDH releases in culture medium were detected using LDH kit (Nanjing Jiancheng Bioengineering Institute, China) strictly according to the manufacturer’s instructions. Detailed steps was performed the same as our previous study [35]. Protein levels were detected to normalize the results by using the BCA protein assay kit (Multi Sciences, China).

### Enzyme-linked immunosorbent assay (ELISA)

The cell culture medium were collected and centrifuged at 12000 rpm for 5 min. The supernatants were transferred to clean 1.5 mL EP tubes and stored at – 80 °C. IL-1β and IL-18 levels in the cell culture medium were measured by ELISA kits (Multi Sciences, China) strictly according to the manufacturer’s instruction [45].

Total protein in the prefrontal cortex, hippocampus were extracted by using lysis buffer which contains RIPA buffer (CWBIO, China), protease inhibitor (Roche, USA). IL-1β and IL-18 levels in these samples were measured by ELISA kits (Elabscience, China) strictly according to the manufacturer’s instruction and previous studies [46, 47]. Protein levels were detected to normalize the results by using BCA protein assay kit.

### Quantitative real-time PCR (qPCR)

Total RNA was isolated from BV2 cells by using a total RNA extraction kit (Promega, China) according to the manufacturer’s protocol. RNA (1 μg) was reverse transcribed into cDNA using a reverse transcription kit (Promega, USA). Subsequently, the qPCR amplification was performed using 2 μL of cDNA and real-time Fluorescence quantitative PCR instrument (Applied Biosystems, USA) according to the manufacturer’s protocol (Promega, USA). Cycling condition: Stage 1:95 °C, 10min; Stage 2:95 °C, 15 s, 60 °C, 1 min, 40 cycles; dissociation stage: 95 °C, 15 s, 60 °C, 15 s, 95 °C, 15 s. The fold change of each gene expression relative to β-actin was determined by the comparative threshold cycle method. Primer sequences (Sangon Biotech, China) are listed in Table 1.

**Table 1:** Primer sequences used in the qPCR

Gene	Primer sequences
IL-1β	Forward:5′-CCAGGATGAGGACATGAGCA-3′
	Reverse:5′-CGGAGCCTGTAGTGCAGTTG-3′
IL-18	Forward:5′-GACTCTTGCGTCAACTTCAAGG-3′
	Reverse:5′-GTTGTCTGATTCCAGGTCTCCA-3′
β-actin	Forward:5′-GTGCTATGTTGCTCTAGACTTCG-3′
	Reverse:5′-ATGCCACAGGATTCCATACC-3′

## Western blot

Total protein in the basal ganglia, prefrontal cortex, hippocampus, and BV2 cells were extracted by using lysis buffer which contains RIPA buffer (CWBIO, China), protease inhibitor (Roche, USA), and phosphatase inhibitor (Roche, USA). BCA protein assay kit was used to detect the protein levels of all samples. The protein samples (30 μg) were separated by 6–12% sodium dodecyl sulfate-polyacrylamide (SDS-PAGE) and transferred to a polyvinylidene fluoride (PVDF) membrane (0.22 μm, Roche, USA). After blocking with 5% bovine serum albumin (Beyotime, China) for 1 h, the membranes were incubated with primary antibodies at 4 °C overnight. The antibodies be used in the present study are listed as follows: Rabbit anti-β-actin (CST, #4970), Rabbit anti-GAPDH (CST, #5174), Rabbit anti-cleaved caspase1(CST, #67314), Rabbit anti-NLRP3 (abcam, ab210491), Rabbit anti-p65 (CST, #8242), Rabbit anti-phosphorylation of p65 (p-p65) (CST, #3033), Mouse anti-IκB (CST, #4814), Rabbit anti-phosphorylation of IκB (p-IκB) (CST, #2859), Rabbit anti-IL-1β (abcam, ab9722), Rabbit anti-IL-18 (abcam, 191860), and Rabbit anti-CD11b (Thermo, PA5-79532). Subsequently, the membranes were washed with TBST and incubated with anti-rabbit IgG (CST, #7074) or anti-mouse IgG (CST, #7076) at room temperature for 1 h. Finally, protein bands were visualized by using an enhanced chemiluminescence system (Thermo, USA) and qualified using Image J.

### Statistical analysis

All data were presented as the mean ± S.D. and analyzed using SPSS statistical software (version 23.0, IBM). Repeated one-way analysis of variance (ANOVA) was used to analyze the results of the Morris water maze test. Other data was analyzed by one-way analysis of variance. ANOVA followed by least significant difference (LSD) tests was used for multiple comparisons. The Games-Howell correction test was used, when equal variance not assumed. Statistical significances were considered as *p* values < 0.05.

## Results

### PAS-Na enhances the learning ability of Mn-exposed rats

**Fig. 1:**
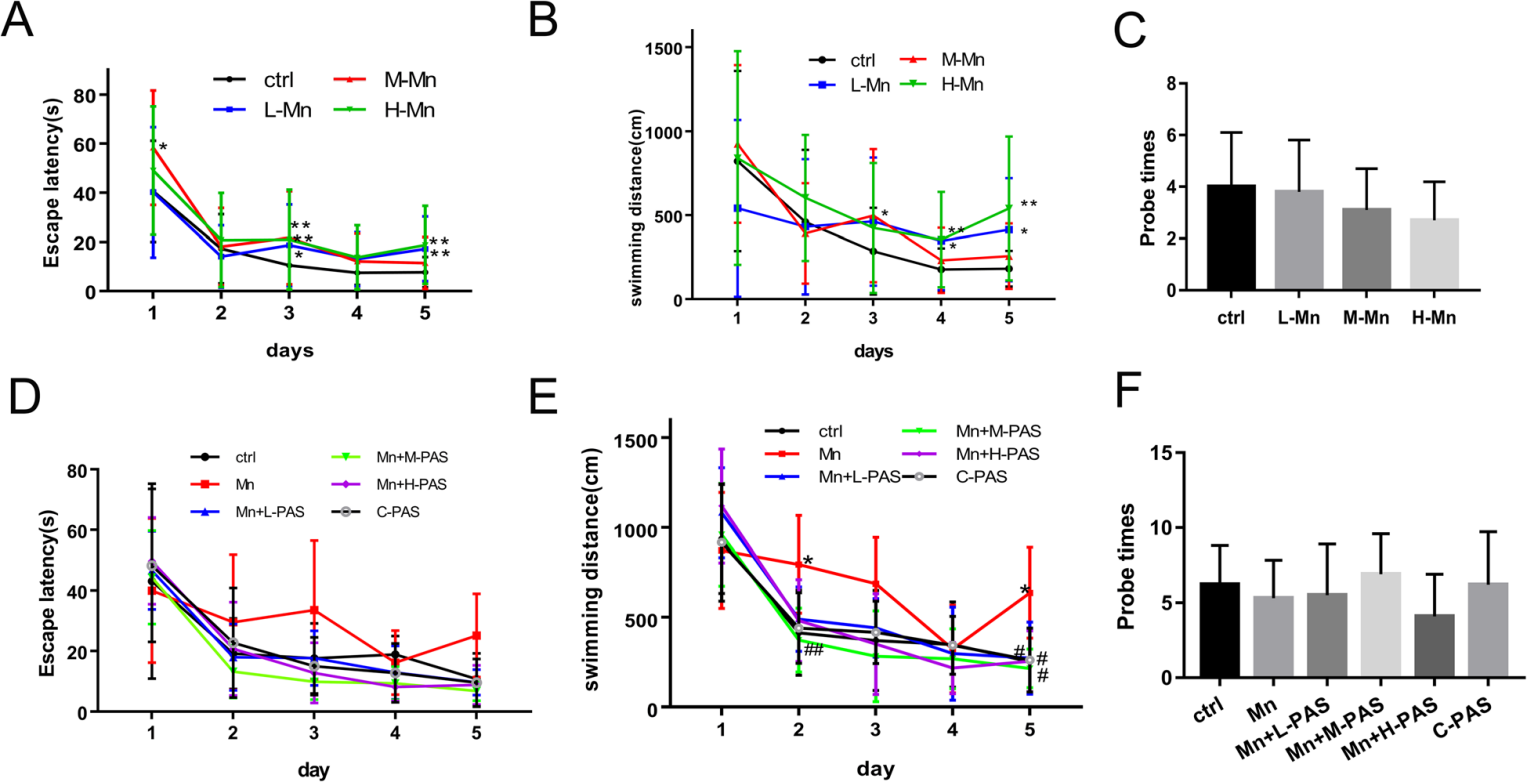
PAS-Na enhanced the learning ability of Mn-exposed rats. Experimental period of 8 weeks: the rats were treated with 5, 10, and 20 mg/kg MnCl_2_ for 8 weeks (**a**–**c**). Experimental period of 14 weeks: the rats were treated with 20 mg/kg MnCl_2_ for 8 weeks and then treated with 100, 200, and 300 mg/kg PAS-Na for an additional 6 weeks (**d**–**f**). Escape latency (**a**, **d**), swimming distance (**b**, **e**), and number of platform crosses (Probe times) (**c**, **f**) of rats were tested by Morris water maze. Data are presented as mean ± SD (*n* = 10 per group). *p < 0.05: significant as compared to the control group; ^#^*p* < 0.05 and ^##^*p* < 0.01: significant as compared to Mn-treated group

Accumulation of Mn in the brain could cause neurological dysfunction, including learning and memory disorders, bradykinesia, etc. Our previous study showed that Mn levels in the striatum and globus pallidus of rats were dramatically increased after 15 mg/kg MnCl_2_ treatment for 3 weeks compared to the control group (1.53 ± 0.04 vs 0.92 ± 0.02 and 1.47 ± 0.03 vs 0.87 ± 0.07, respectively) [48]. To make the behavioral changes induced by Mn valid, rats were applied in this study and the learning and memory capacity were tested using Morris water maze. The escaping latency and swimming distance of Mn-exposed rats were significantly increased after exposure to MnCl_2_ for 8 weeks compared to the control group (*p* < 0.05 or 0.01, Fig. 1a, b). The swimming distance still increased after exposure to 20 mg/kg MnCl_2_ for 8 weeks and followed by 6 weeks of no Mn exposure (*p* < 0.01, Fig. 1e). After treatment with PAS-Na, the swimming distances were shorter on the 2nd and 5th training days compared to the Mn-exposed group (*p* < 0.05 or 0.01, Fig. 1e). However, the frequency difference of probe times between the Mn-treated group and the control group was not significant (*p* > 0.05, Fig. 1c, f).

### PAS-Na inhibits Mn-induced oxidative stress in BV2 cells

**Fig. 2:**
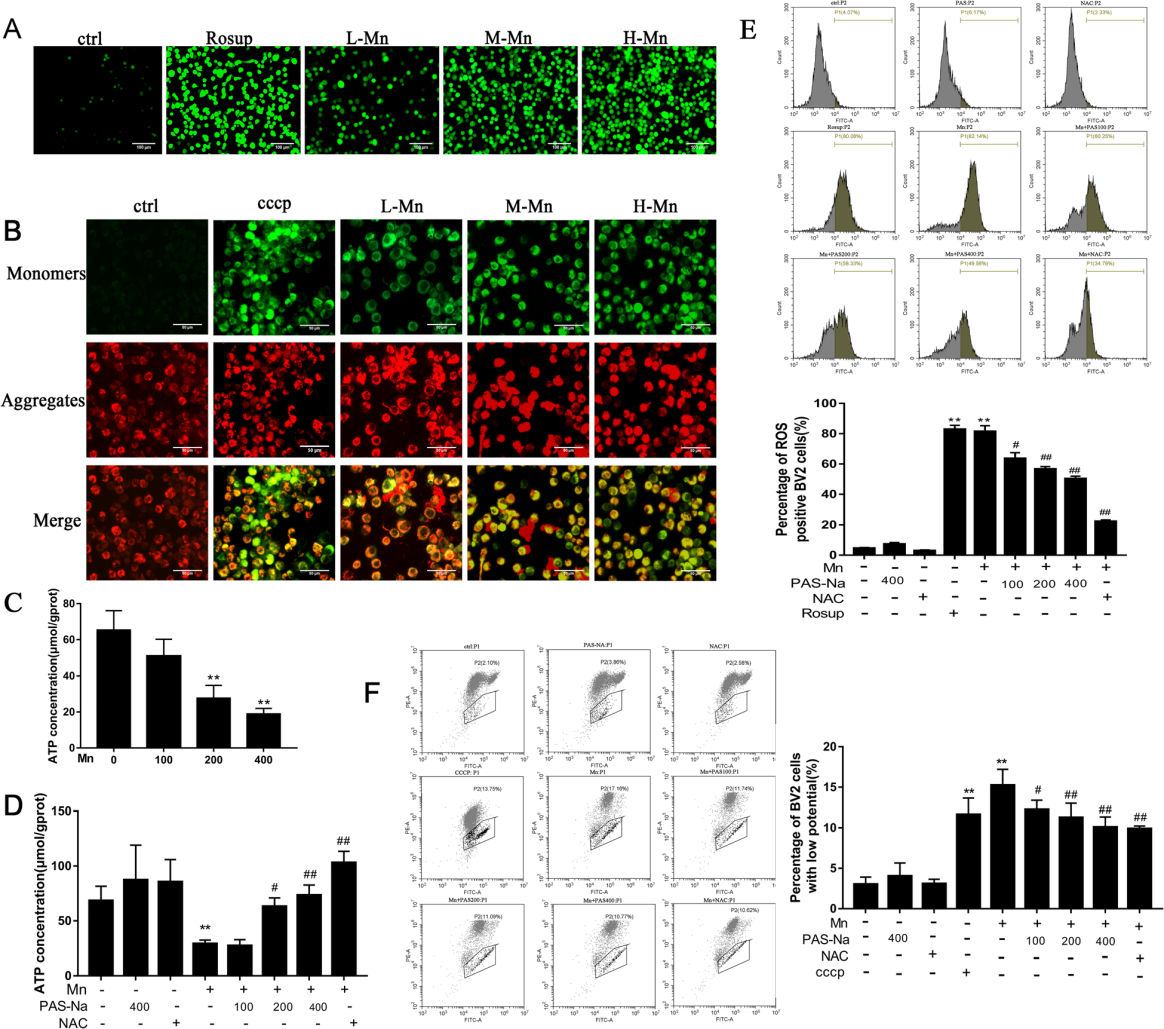
PAS-Na inhibited Mn-induced oxidative stress in BV2 cells. **a**–**c** BV2 cells were treated with 100, 200, and 400 μmol/L MnCl_2_ for 24 h, respectively. Cells in positive control group were treated with rosup or CCCP (**a**, **e**) (a compound mixture that was provided by the manufacturer) for 1 h after normal medium cultured for 23 h. **a** ROS in BV2 cells was detected by DCFH-DA kit. After DCFH-DA treatment, ROS shows green fluorescence. Scale bars: 100 μm. **b** Mt∆Ψm of BV2 cells was determined by the lipophilic cationic probe JC-1. When the mt∆Ψm is high, JC-1 gathers in the mitochondrial matrix to form J-aggregates and produces red fluorescence. On the contrary, JC-1 is presented as a monomer and produces green fluorescence. Scale bars: 50 μm. **c** Mn decreased intracellular ATP concentration in BV2 cells. **d**–**f** BV2 cells were treated with 200 μmol/L MnCl_2_ for 24 h, following treatment with 100, 200, and 400 μmol/L PAS-Na and 50 umol/L NAC for 24 h, respectively. **d** PAS-Na recovered the ATP levels in Mn-treated BV2 cells. **e** Flow cytometry analysis of intracellular ROS in BV2 cells. **f** Flow cytometry analysis of intracellular the mt∆Ψm change of BV2 cells. All tests were repeated independently three times. Data are presented as mean ± SD. *p < 0.05 and **p < 0.01: significant as compared to the control group; #*p* < 0.05 and ##*p* < 0.01: significant as compared to Mn-treated group

To detect the oxidative damage caused by Mn and the protective effect of PAS-Na, we measured ROS production, mt∆Ψm changes and ATP levels in BV2 cells, using NAC (an antioxidant, Beyotime, China) as a positive control [49]. The fluorescent images showed that ROS levels in all doses of Mn-treated groups were higher than those in the controls (Fig. 2a). Besides, after BV2 cells were treated with Mn for 24 h, the mt∆Ψm of cells decreased significantly, compared to the control group (Fig. 2b). ATP production was also decreased in the 200 and 400 μmol/L Mn-treated groups (*p* < 0.01, Fig.2c), which might be due to the decrease of mt∆Ψm and blocked electron transport. However, PAS-Na and NAC treatment decreased the intracellular ROS and recovered the loss of mt∆Ψm in Mn-treated BV2 cells compared to the Mn-treated group (*p* < 0.05 or *p* < 0.01, Fig. 2e, f). Subsequently, ATP levels in the Mn-treated BV2 cells were recovered as compared with the Mn-treated group (*p* < 0.05 or *p* < 0.01, Fig. 2d). Administration of PAS-Na and NAC alone has no effects on the intracellular ROS, mt∆Ψm, and ATP in BV2 cells (*p* > 0.05, Fig. 2d–f). Our previous study confirmed that PAS-Na could inhibit Mn-induced oxidative stress by increasing the activity of primary anti-oxidant enzymes glutathione peroxidase (GSH-Px) and catalase (CAT) [35]. These results indicated that PAS-Na could effectively resist the oxidative damage caused by Mn.

### PAS-Na inhibits Mn-induced NF-κB pathway activation by suppressing oxidative stress in BV2 cells

**Fig. 3:**
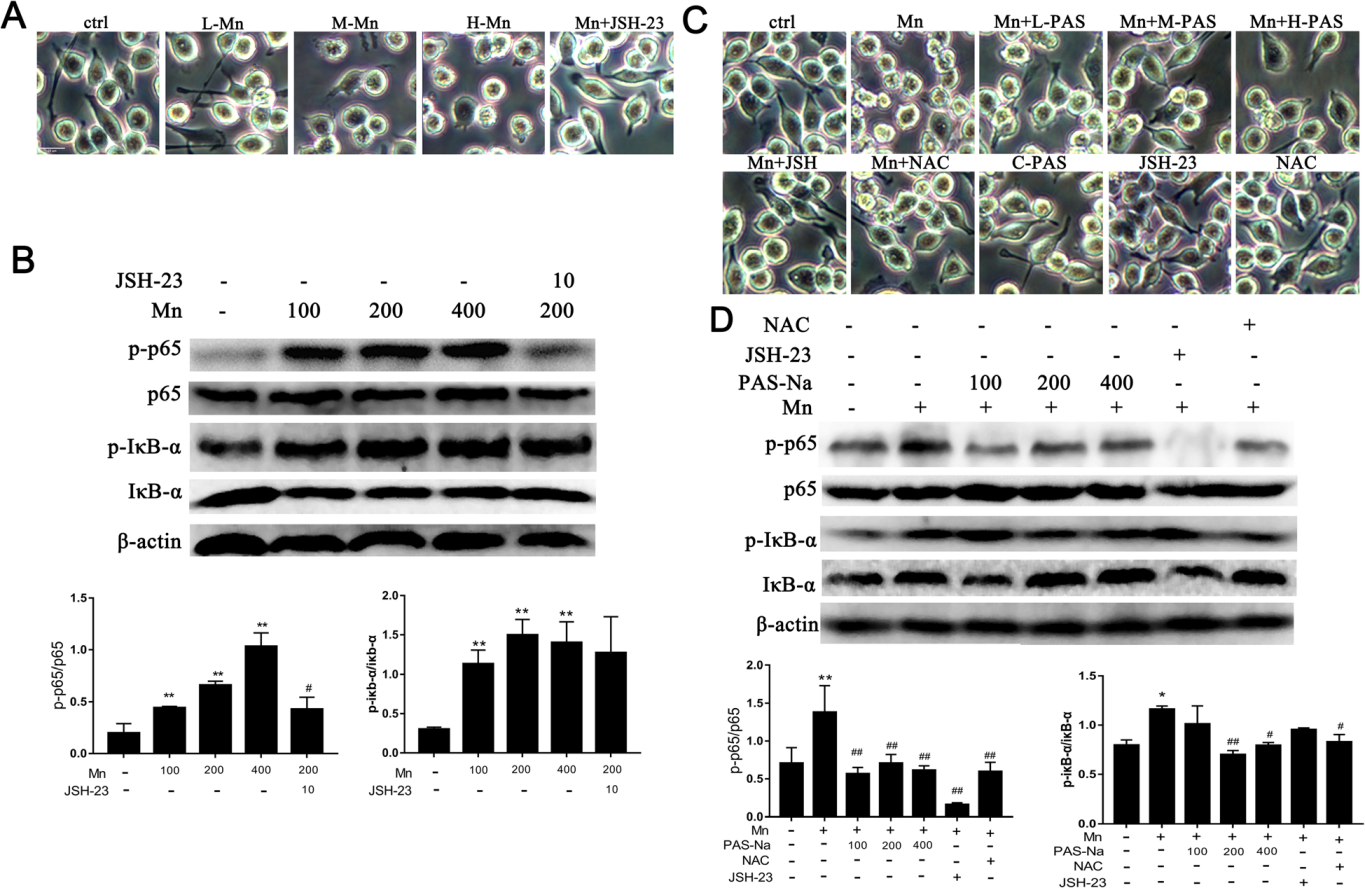
PAS-Na inhibited Mn-induced NF-κb pathway activation by suppressing oxidative stress in BV2 cells. **a**, **b** BV2 cells were treated with 100, 200, and 400 μmol/L MnCl_2_ or 200 μmol/L MnCl_2_ + 10 μmol/L JSH-23 (L-, M-, H-Mn, Mn + JSH-23) for 24 h, respectively. **a** Morphology of BV2 cell. Scale bars: 20μm. **b** The protein expressions of p-p65, p65, p-IκB-α, and IκB-α in BV2 cells were detected by western blot. **c**, **d** BV2 cells were treated with 200 μmol/L MnCl_2_ or 200 μmol/L MnCl2 + 10 μmol/L JSH-23 for 24 h, following treatment with 100, 200, and 400 μmol/L PAS-Na and 50 μmol/L NAC for 24 h, respectively. **c** Morphology of BV2 cell. Scale bars: 20μm. (D) The protein expressions of p-p65, p65, p-IκB-α, and IκB-α in BV2 cells were detected by western blot. The protein expression was normalized by β-actin or corresponding total protein content. All tests were repeated independently three times. Data are presented as mean ± SD.*p < 0.05 and **p < 0.01: significant as compared to the control group; #*p* < 0.05 and ##*p* < 0.01: significant as compared to corresponding Mn-treated group

Oxidative stress can activate inflammatory pathways such as NF-κB, causing inflammation. NAC and JSH-23 (an inhibitor of transcriptional activity of NF-κB, Selleck Chemicals, USA) were used here. As shown in Fig. 3a, Mn-treated BV2 cells had fewer branches and cell membrane was destroyed, displayed a typical feature of pyroptosis membrane rupture [50]. As the Mn treatment dose increases, this morphological change becomes more severe. We also found that Mn could activate the NF-κB pathway by increasing the phosphorylation of p65 and IkB-α in BV2 cells (Fig. 3b). Next, we explored whether PAS-Na could attenuate the activation of NF-κB pathway by inhibiting oxidative stress. PAS-Na and NAC treatment clearly restored cell morphology as compared with the Mn-treated group (Fig. 3c). Addition of PAS-Na and NAC alone hardly affected BV2 cell morphology. However, PAS-Na reduces the p-p65 and P-IκB-α caused by Mn, and its effect is similar to NAC, especially the phosphorylation of p65 when treated with 100 μmol/LPAS-Na and IκB-α when treated with 200 μmol/LPAS-Na (*p* < 0.05 or *p* < 0.01, Fig. 3d). Collectively, this confirmed that PAS-Na could inhibit the activation of NF-κB by inhibiting Mn-induced oxidative stress.

### PAS-Na mitigates Mn-induced BV2 cells activation and microglia proliferation by inhibiting NF-κB pathway activation

**Fig. 4:**
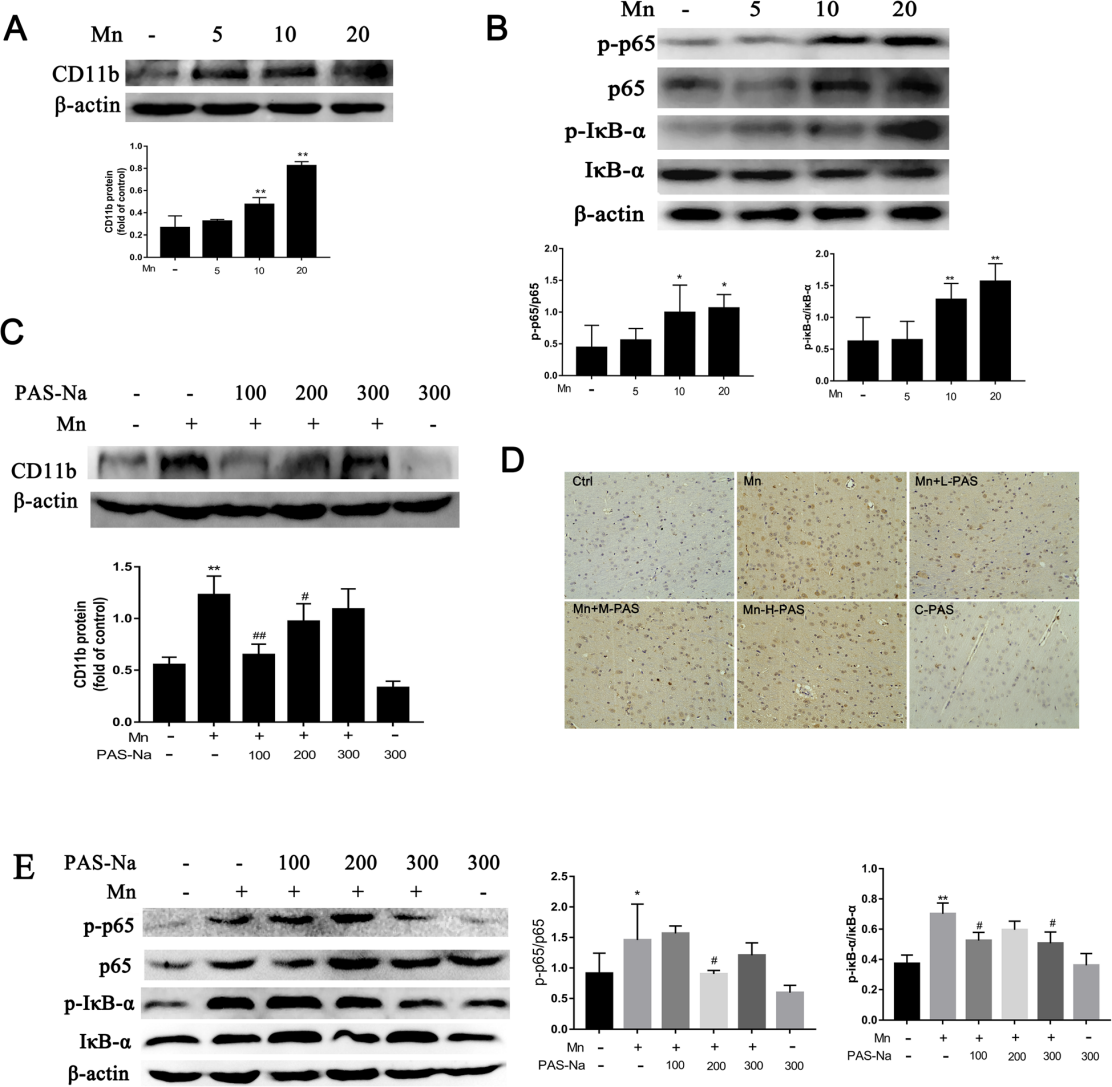
PAS-Na mitigates Mn-induced BV2 cells activation and microglia proliferation in basal ganglia of rats by inhibited NF-κB pathway activation. **a**, **b** The rats were treated with 5, 10, and 20 mg/kg MnCl_2_ for 8 weeks. **a** The protein expressions of CD11b in the basal ganglia of rats was analyzed by western blot (*n* = 4 per group). **b** The protein expressions of p-p65, p65, p-IκB-α, and IκB-α in the basal ganglia of rats was detected by western blot (*n* = 4 per group). **c**–**d** The rats were treated with 20 mg/kg MnCl_2_ for 8 weeks and then treated with 100, 200, and 300 mg/kg PAS-Na for an additional 6 weeks. **c** The protein expression of CD11b in the basal ganglia of rats were analyzed by western blot (*n* = 4 per group). **d** Immunohistochemical results of CD11b in the basal ganglia of rats (*n* = 3 per group). Scale bars: 100 μm. **e** The protein expressions of p-p65, p65, p-IκB-α, and IκB-α in the basal ganglia of rats were detected by western blot (*n* = 4 per group). The protein expression was normalized by β-actin or corresponding total protein content. Data are presented as mean ± SD.*p < 0.05 and **p < 0.01: significant as compared to the control group; #*p* < 0.05 and ##*p* < 0.01: significant as compared to corresponding Mn-treated group

In order to clarify the connections among Mn-induced microglia activation, NF-κB pathway and the effect of PAS-Na intervention, we used JSH-23 as a positive control [51]. Our results showed that P65 could enter the nucleus in Mn-treated BV2 cells, which can be inhibited by JSH-23 (Figure S1). The expression of p-p65 and p-IκB-α in Mn-treated BV2 cells (Fig. 3b) and in the basal ganglia of Mn-exposed rats were significantly increased (*p* < 0.05 or *p* < 0.01, Fig. 4b). Nonetheless, as compared with the Mn-treated group, the expression of p-p65 in PAS-Na treatment groups and JSH-23 treatment group were significantly decreased, while the decrease in JSH-23 treatment was more robust (*p* < 0.01, Fig. 3d). Different doses of PAS-Na and JSH-23 treatment also restored cell morphology as compared with the Mn-treated group (Fig. 3c), while the JSH-23 alone hardly affected. This data suggested the specificity of PAS-Na in protecting Mn-induced toxicity. CD11b is specifically expressed in microglia of brain which often used to identify activated microglia [52–54]. The protein expression of CD11b in the basal ganglia of rats was significantly increased after exposure to 10 and 20 mg/kg MnCl_2_ (*p* < 0.01, Fig. 4a). Furthermore, we used in vivo studies to further verify our findings and got similar results that the expression of CD11b, p-IκB-α, and p-p65 in the basal ganglia of PAS-Na treatment groups were significantly decreased compared to the Mn-exposed group (*p* < 0.01, Fig. 4c, e, respectively). And p-p65 in the prefrontal cortex and hippocampus of PAS-Na treatment groups were significantly decreased compared to the Mn-exposed group (*p* < 0.001, Fig. 7a). Besides, compared with the Mn-exposed group, immunohistochemical results of CD11b showed that the number of CD11b-positive cells was significantly reduced after PAS-Na intervention, especially in the Mn + L-PAS and Mn + M-PAS groups (Fig. 4d). These results indicated that PAS-Na might alleviate Mn-induced BV2 cells activation and microglia proliferation in basal ganglia of rats by inhibiting the activation NF-κB pathway.

### PAS-Na inhibits Mn-induced NLRP3 inflammasome-dependent pyroptosis by inhibiting NF-κB pathway activation and oxidative stress

**Fig. 5:**
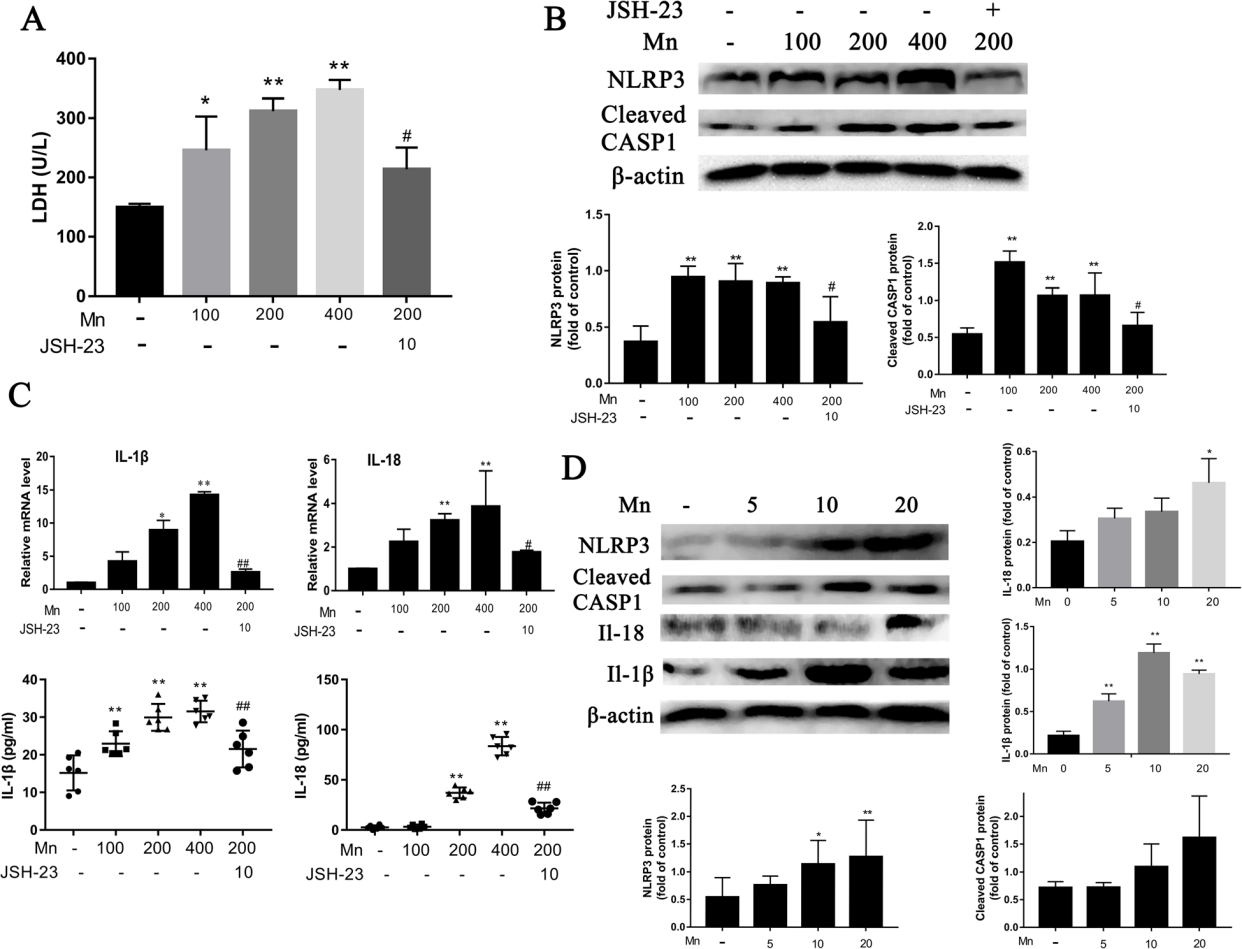
Mn activated the NLRP3-CASP1 inflammasome-dependent pyroptosis both in BV2 cell and in the basal ganglia of rats. **a**–**c** BV2 cells were treated with 100, 200, and 400 μmol/L MnCl_2_ or 200 μmol/L MnCl_2_ + 10 μmol/L JSH-23 for 24 h. **a** Relative release of LDH in BV2 cells culture medium. **b** The protein expressions of NLRP3, cleaved-caspase1 in BV2 cells were detected by western blot. **c** The IL-1β and IL-18 mRNA expression in BV2 cells were detected by qPCR. The IL-1β and IL-18 level in the culture medium were detected by ELISA. **d** The rats were treated with 5, 10, and 20 mg/kg MnCl_2_ for 8 weeks. The protein expressions of NLRP3, cleaved-caspase1, IL-1β, and IL-18 in the basal ganglia of rats were detected by western blot (*n* = 4 per group). The protein expression was normalized by β-actin. All tests were repeated independently three times. Data are presented as mean ± SD.*p < 0.05 and **p < 0.01: significant as compared to the control group; #*p* < 0.05 and ##*p* < 0.01: significant as compared to corresponding Mn-treated group

To investigate whether PAS-Na inhibits Mn-induced NLRP3 inflammasome-dependent pyroptosis, in vitro and in vivo studies were conducted simultaneously. Measurement of LDH release was performed to assess the pyroptosis (Schneider et al. 2017). Mn treatment increased the LDH release in culture medium in a dose-dependent manner, as compared with the control (*r* = 0.952, *p* < 0.05, Fig. 5a). ROS and mitochondrial dysfunction can activate NLRP3. In this study, the expression of NLRP3 and cleaved caspase-1(Cleaved CASP1) were significantly increased after Mn treatment in BV2 cells and in the basal ganglia of rats (*p* < 0.05 and *p* < 0.01, respectively, Fig. 5b, d). Concomitantly, both the IL-1β and IL-18 mRNA and their secretion levels in Mn-treated BV2cells or culture medium were increased dramatically compared to control (*p* < 0.01, Fig. 5c). The IL-1β and IL-18 protein expression was also significantly increased in the basal ganglia of Mn-exposed rats as compared with those in control (*p* < 0.01, Fig. 5d). However, LDH relative release, the expression of NLRP3 and cleaved CASP1 protein and both the mRNA and secreted protein of IL-1β and IL-18 level were significantly reduced in JSH-23 treatment groups compared to the corresponding Mn-treated group (*p* < 0.05 and *p* < 0.01, respectively, Fig. 5a–c), which implied that NF-κB played a crucial role in NLRP3 inflammasome-dependent pyroptosis.

**Fig. 6:**
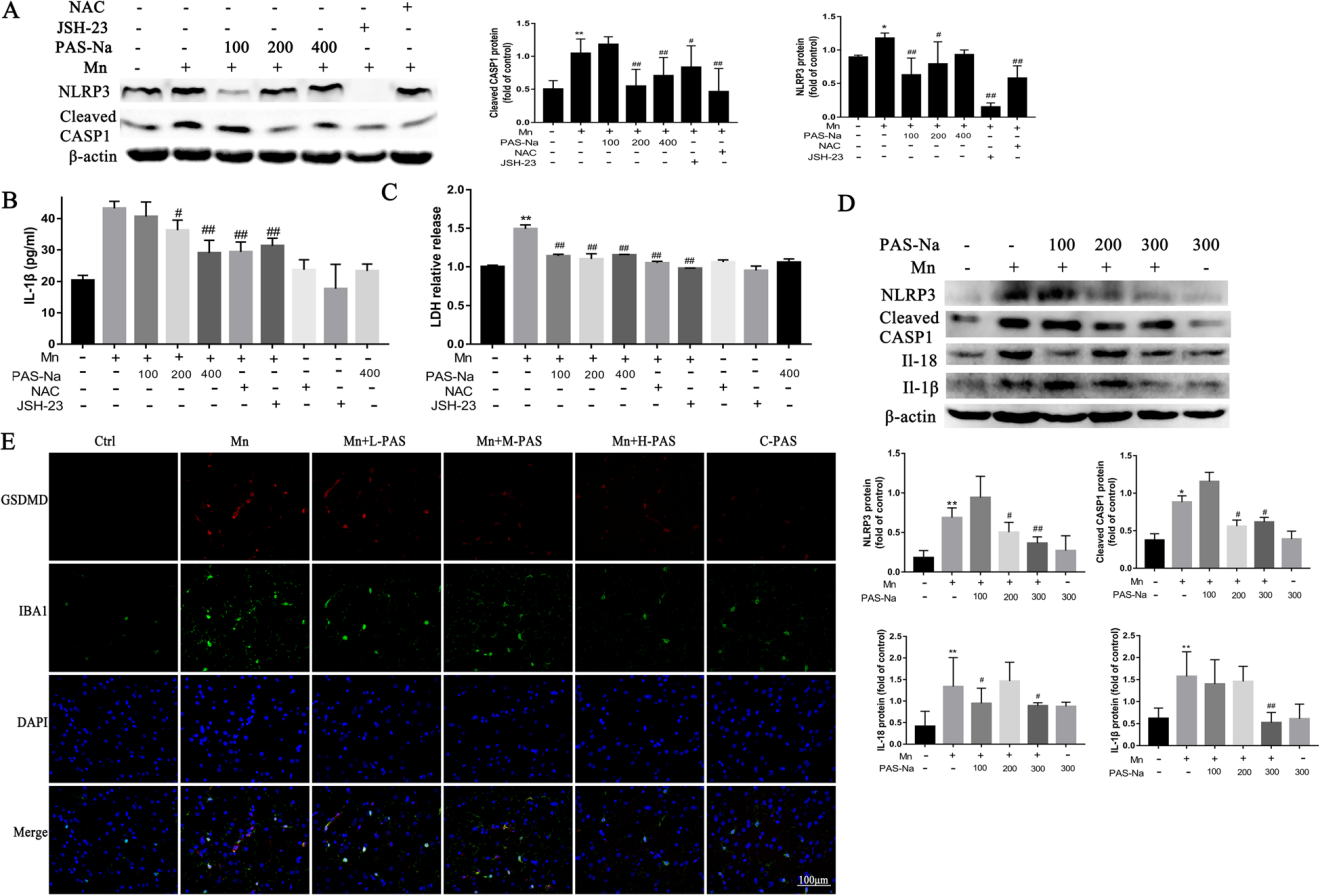
PAS-Na inhibited Mn-induced NLRP3 inflammasome-dependent pyroptosis by inhibited NF-κB pathway activation and anti-oxidative stress. **a**–**c** BV2 cells were treated with 200 μmol/L MnCl_2_ or 200 μmol/L MnCl2 + 10 μmol/L JSH-23 for 24 h, following treatment with 100, 200, and 400 μmol/L PAS-Na and 50 μmol/L NAC for 24 h, respectively. **a** The protein expressions of NLRP3, cleaved-caspase1 in BV2 cells were detected by western blot. **b** Levels of IL1β in the BV2 cell culture supernatants were measured by ELISA. **c** Relative release of LDH in BV2 cells culture medium. **d**, **e** the rats were treated with 20 mg/kg MnCl_2_ for 8 weeks and then treated with 100, 200, and 300 mg/kg PAS-Na for an additional 6 weeks. **d** The protein expressions of NLRP3, cleaved-caspase1, IL-1β, and IL-18 in the basal ganglia of rats were detected by western blot (*n* = 4 per group). **e** The basal ganglia sections of rats were stained for IBA1 as the microglia marker (green staining), GSDMD (red staining). Nuclei were stained with DAPI. Bar: 100 μm. The protein expression was normalized by β-actin. All tests were repeated independently three times. Data are presented as mean ± SD.*p < 0.05 and **p < 0.01: significant as compared to the control group; #*p* < 0.05 and ##*p* < 0.01: significant as compared to corresponding Mn-treated group

**Fig. 7:**
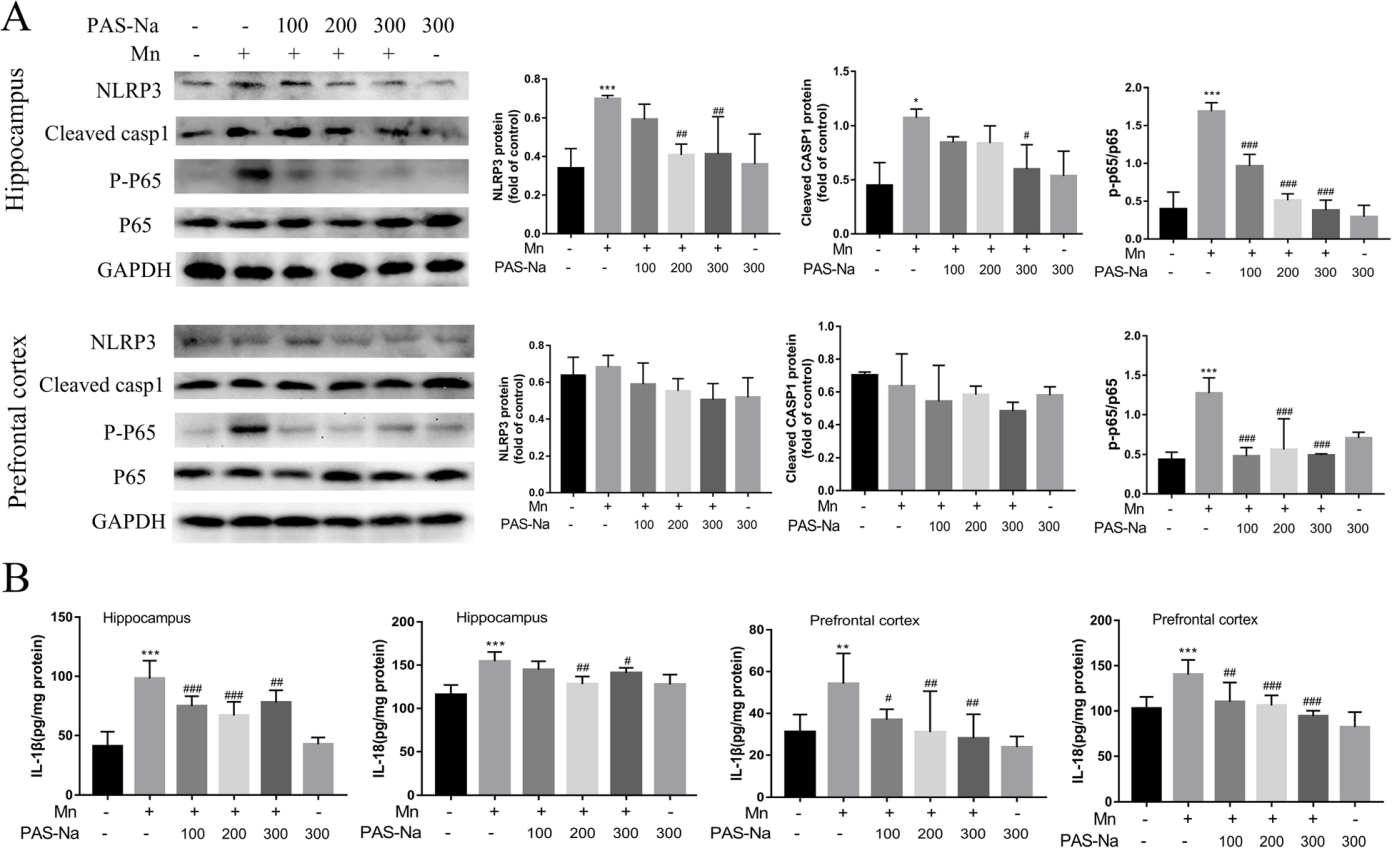
PAS-Na inhibited Mn-induced NLRP3 inflammasome-dependent pyroptosis. **a** The protein expressions of NLRP3, cleaved-caspase1, P-P65, and P65 in the prefrontal cortex and hippocampus of rats were detected by western blot. b The IL-1β and IL-18 level in the prefrontal cortex and hippocampus of rats were measured by ELISA. The protein expression was normalized by GAPDH. All tests were repeated independently three times. Data are presented as mean ± SD. **p < 0.01 and ***p < 0.001: significant as compared to the control group; #*p* < 0.05, ##*p* < 0.01, and ###*p* < 0.001: significant as compared to corresponding Mn-treated group

As the above results confirmed that PAS-Na could inhibit Mn-induced oxidative stress and NF-κB activation, we further investigated whether it could prevent Mn-induced NLRP3 inflammasome-dependent pyroptosis. After treatment with PAS-Na for 24 h, the relative release of LDH (Fig. 6c) and the secretion of IL-1β (Fig. 6b) in the medium also decreased significantly. The expression of NLRP3 and cleaved CASP1 in the control group markedly decreased as compared to the Mn-treated BV2 cells (*p* < 0.05 or *p* < 0.01, Fig. 6a). The decrease in the JSH-23 treatment group was more robust after treatment with JSH-23 (*p* < 0.05 or *p* < 0.01, Fig. 6b, c). The NLRP3 and cleaved CASP1 expressions in the NAC treatment group also significantly decreased as compared with the Mn-treated group, especially cleared CASP1 (*p* < 0.01, Fig. 6 b). The relative release of LDH and the secretion of IL-1β in the medium also had a pronounced decrease after NAC treatment for 24 h (*p* < 0.01, Fig. 6a, c). Moreover, in vivo studies have shown similar results that PAS-Na could antagonize the increase of NLRP3, cleaved CASP1, IL-18, and IL-1β protein expressions induced by Mn in the basal ganglia (*p* < 0.05 or *p* < 0.01, Fig. 6d) and hippocampus (*p* < 0.05, *p* < 0.01, or *p* < 0.001, Fig. 7a, b) of rats. The expressions of NLRP3 and cleaved CASP1 did not increase significantly in the prefrontal cortex as compared with the Mn-treated group (*p* > 0.05, Fig. 7a). GSDMD is a pyroptosis effector that is widely used to evaluate the occurrence of pyroptosis (Schneider et al. 2017). In this study, immunofluorescence results showed that Mn treatment increased the number of activated microglia, which stained positively for GSDMD (Fig.6e). However, PAS-Na inhibited this increase (Fig. 6e). These results implied that PAS-Na might inhibit Mn-induced NLRP3 inflammasome-dependent pyroptosis by inhibiting the activation of NF-κB pathway and oxidative stress.

## Discussion

Excessive exposure to Mn can cause manganism, although Mn is an essential trace element for the human. As excessive Mn mainly accumulates in the basal ganglia, Mn poisoning can cause motor and learning disabilities, which shares clinical presentation with Parkinson's disease (PD) [55–57]. Many studies have confirmed that the activation of microglia and NLRP3-inflammasome may be involved in Mn-induced inflammation [27, 28]. Our previous studies have shown that PAS-Na can alleviate Mn-induced inflammation [25, 32]. In this study, BV2 cells and male SD rats were used to evaluate whether PAS-Na could attenuate Mn-induced NLRP3 inflammasome-dependent pyroptosis.

Microglia, a type of glial cells that acts as macrophage in the brain, is the first and primary immune defense in the CNS [58, 59]. Various clinical and neuropathologic studies have shown that the activation of microglial cells is involved in the pathogenesis of neurodegenerative diseases [44, 60, 61]. It is an essential source of pro-inflammatory factors and oxidative stress, such as TNF, interleukin and other neurotoxic substances [58, 59]. Many studies showed that heavy metals could induce neurotoxicity via activating the microglial cells, especially Mn [22, 23, 62]. In vitro study demonstrated that Mn and/or LPS can activate BV2 cells via activating MAP kinase, PI3K/Akt pathways and inflammatory process [63]. As above, we selected BV2 cells for in vitro research. It is worth noting that microglia in the globus pallidus external segment (GPe), and globus pallidus internal segment (GPi) of workers chronically exposed to Mn were increased by 53% and 38%, respectively, as compared with those in non-Mn exposed workers [7]. An in vivo studies have shown that microglia in the hippocampus of Mn-exposed rats were activated displaying a dramatic change in morphology and staining positively for NLRP3 [28]. Besides, Chronic Mn exposure can result in apoptosis of central nerve cells in rats’ hippocampus, and upregulate Hsp70 transcription and translation. Our results show that Mn could promote the activation the NF-κB inflammation pathway and/or proliferation of microglia and in the basal ganglia, hippocampus and prefrontal cortex of Mn-exposed rats. Therefore, further research on the neurotoxicity of Mn in microglia and parallel studies on any neuroprotective activity of PAS-Na will be valuable.

Oxidative stress, which refers to the imbalance between ROS and antioxidant functions in the body, plays a critical role in the CNS. Various indicators of oxidative stress have been documented in neurodegenerative diseases [64]. Oxidative stress has been shown to participate in Mn-induced neurotoxicity [65, 66]. For example, excessive Mn exposure induced oxidative damage by down-regulating the protein expressions of the neuronal excitatory amino acid carrier 1 (EAAC1) and cystine/glutamate exchanger (xCT) to restrain glutathione synthesis in the striatum of mice [67]. Mitochondria play a pivotal role in normal biological functions by maintaining homeostasis of ATP, metal ions, and ROS [68, 69]. The neurotoxic effects of Mn interrupt this homeostasis, increasing ROS generation and decreasing the ATP level in BV2 cells. In the normal tricarboxylic acid (ATC) cycle, electrons are transferred to O_2_ and eventually form H_2_O, which is regulated by the redox systems of glutathione. In the process of respiration and oxidation, mitochondria store the energy generated in the inner mitochondrial membrane with electrochemical potential energy, which causes an asymmetric distribution of proton and other ion concentrations on both sides of the inner membrane to form mt∆Ψm [70, 71]. More importantly, protons act through the respiratory enzyme complex to form H_2_O and release ATP in the mitochondria electron transfer chain (ETC) protein complexes [68]. However, mitochondrial damage could lead to the collapse of the mt∆Ψm and obstacle production [72]. Both the deficiency and excessive accumulations of one or multiple metals may cause mitochondrial dysfunction [73]. In this study, Mn treatment led to the collapse of the mt∆Ψm, suggesting that the metal balance in the mitochondria was disrupted. Future experiments need to be done to detect metal levels in mitochondria to support this conclusion directly. Research has shown that PAS-Na can inhibit Mn-induced oxidative stress by increasing the activities of primary anti-oxidant enzymes glutathione peroxidase (GSH-Px) and catalase (CAT) (Li et al. 2016). Our results show that PAS-Na could alleviate Mn-induced oxidative stress damage by inhibiting ROS generation, increasing mitochondrial membrane potential and ATP levels. These results indicate that PAS-Na can effectively resist Mn-induced oxidative damage.

NF-κB, a major transcription factor in immune regulation, is widely expressed in biological systems [10]. Inhibitory proteins of the κb family (IκB) plays a valuable role in regulating the normal balance of NF-κB [10]. The activation of NF-κB induced by phosphorylated IκB might interrupt the balance of transcription genes to activate NLRP3 and pro-IL-1β [15, 74]. An earlier study indicated that the p65 subunit plays a significant role in maintaining the formation of excitatory synapses, dendritic spines, the morphology of mouse hippocampal neurons [75], and astrocytic process plasticity of mouse mediobasal hypothalamus [76]. NF-κB also takes part in regulating the inflammatory response in microglia [77]. Recently, NF-κB signaling pathway has been shown to play a major role in Mn-induced promotion of inflammatory response and apoptosis. For example, an in vitro study demonstrated that Mn activated NF-κB by interacting with PI3K/Akt pathway in astrocytes [78] and increased NF-κB DNA binding activity in PC12 cells [79]. Interestingly, ROS is critical for nuclear reprogramming and the activation of NF-κB signaling [80, 81]. The present results show that PAS-Na can inhibit Mn-induced NF-κB activity, which is similar to that of antioxidant NAC. These findings suggest PAS-Na may inhibit Mn-induced NF-κB pathway activation through the suppression of oxidative stress.

Pyroptosis, a new way of programmed cell death, is characterized by reliance on caspase-1 accompanied by the release of a large amount of pro-inflammatory factors. It is worth noting that pyroptosis is involved in various neurodegenerative diseases [17, 44, 50]. As known, organisms could sense intracellular and extracellular dangerous signals through Nod-like receptors (NLRs) [82]. The NLRs distinguishes the signals and triggers a signaling cascade leading to pyroptosis and the production of inflammatory cytokine [82, 83]. McKenzie et al. reported that the protein expressions of pyroptosis-related genes were elevated in the microglia cells of multiple sclerosis models, including IL-1β, IL-18, caspase-1, and NLRP3 [44]. Furthermore, mitochondrial dysfunction and excessive ROS generation always accompanied by PD occurrence and development via NLRP3 inflammasome-dependent pyroptosis, and thus promote the secretion of IL-1β and IL-18 [61]. Wang et al. reported that Mn activated the NLRP3 inflammasome pathway via triggering autophagy dysfunction and promoting the secretion of IL-1β and IL-18 [28]. This study also showed that Mn activated the NLRP3 inflammasome and promoted the secretion of IL-1β and IL-18. Excitedly, NF-κB signaling acts as a priming signal to activate the NLRP3 inflammasome pathway and then promotes the secretion of IL-1β [27]. The activations of the NLRP3 inflammasome relies on the ROS generations [30, 31]. Therefore, both oxidative stress and NF-κB activation can be therapeutic targets for the neurotoxicity of Mn. PAS-Na is active in the treatment of manganism. Earlier studies showed that PAS-Na increased the antioxidant enzyme activities of glutathione peroxidase and catalase in Mn-treated primary basal ganglia neurons [35]. Further, previous studies demonstrated that PAS-Na decreased IL-1β and TNF-α levels in the serum of Mn-exposed rats, as well as the brain E2 prostaglandin (PGE2) levels via modulating MAPK pathway [25, 32, 37]. PAS-Na has been shown to partially reverse the learning and memory dysfunction induced by Mn [25, 84]. The present study showed that PAS-Na could antagonize the increase of NLRP3, cleaved CASP1, IL-18, and IL-1β protein expression induced and inhibited the increase of GSDMD-positive cells in the basal ganglia and hippocampus of Mn-exposed rats; the learning ability of these rats was subsequently improved. Summarily, PAS-NA may inhibit Mn-induced NLRP3 inflammasome-dependent pyroptosis by inhibiting NF-κB pathway activation and oxidative stress.

## Conclusion

We provide evidence in this study that exposure to Mn can result in neurotoxicity particularly through ROS increase, mitochondrial dysfunction, ATP level decrease, NF-κB pathway activation, and NLRP3 inflammasome-dependent pyroptosis occurrence. PAS-Na can attenuate Mn-induced activation of NLRP3 inflammasome-dependent pyroptosis by inhibiting NF-κB pathway activation and oxidative stress. These findings provide insight into the therapeutic mechanisms of PAS-Na on Mn-induced neurotoxicity. Future studies incorporating the long-term exposure of Mn at environmentally relevant concentrations are desirable to further understand the therapeutic mechanisms of PAS-Na.

## Supplementary Information

**Additional file 1: Figure S1.** Supplement results.

## Data Availability

The datasets during and/or analyzed during the current study are available from the corresponding author on reasonable request.

## Abbreviations

NLRP3: NOD-like receptor protein 3
PD: Parkinson’s disease
CNS: Central nervous system
IL-1β: Interleukin-1β
ROS: Reactive oxygen species
DAMPs: Diverse damage-associated molecular patterns
GSDMD: Gasdermin D
PAS-Na: Sodium p-aminosalicylic acid
AD: Alzheimer’s disease
MAPK: Mitogen-activated protein kinase
COX-2: Cyclooxygenase-2
NF-κB: Nuclear factor-Kappa B
EAAC1: Excitatory amino acid carrier 1
SDS-PAGE: Sodium dodecyl sulfate-polyacrylamide
PVDF: Polyvinylidene fluoride membrane
