# Supplementary figures and images for: Sodium para-aminosalicylic acid inhibits manganese-induced NLRP3 inflammasome-dependent pyroptosis by inhibiting NF-κB pathway activation and oxidative stress
Source: J Neuroinflammation. 2020 Nov 17;17:343. doi: 10.1186/s12974-020-02018-6 (PMC7670624; doi:10.1186/s12974-020-02018-6)

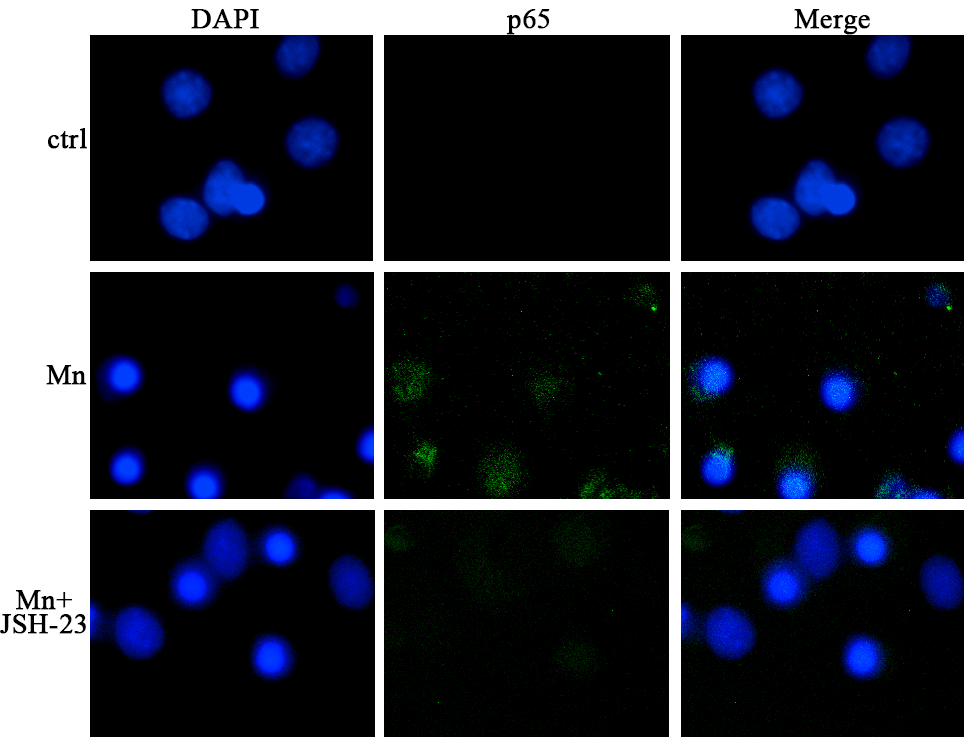

Supplement: Supplementary file 1 — Additional file 1: Figure S1. Supplement results. [file 12974_2020_2018_MOESM1_ESM.tif]
